# The gastrointestinal tract microbiome of Holstein × Angus cross cattle is negatively impacted by the pre-harvest process

**DOI:** 10.1128/aem.02599-24

**Published:** 2025-04-11

**Authors:** M. K. Costello, J. C. McClure, J. A. Brown, R. Amorín de Hegedüs, H. C. Mantovani, S. C. Ricke

**Affiliations:** 1 Department of Animal and Dairy Sciences, University of Wisconsin-Madison 5228, Madison, Wisconsin, USA; 2 United States Department of Agriculture, Dairy Forage Research Center 57844, Madison, Wisconsin, USA; The Pennsylvania State University, University Park, Pennsylvania, USA

**Keywords:** beef, microbiome, GIT, food safety, harvest

## Abstract

Stress during the beef pre-harvest period can induce an inflammatory response and acidotic conditions in the gastrointestinal tract (GIT), which affects the gastrointestinal tract microbiome. The objective of this study was to characterize the status of the GIT microbiome at harvest in beef cattle entering a small USDA processing facility. Nine beef cattle were shipped from a producer in Columbia County, WI, to the USDA processing facility at the University of Wisconsin-Madison and were harvested across four dates. Digesta samples were collected from eight GIT locations: rumen solids, rumen liquids, abomasum, duodenum, jejunum, ileum, cecum, and large intestines. After DNA extraction, the V4 region of the 16S rRNA gene was amplified and sequenced on the Illumina MiSeq platform. Sequences were analyzed for alpha and beta diversity metrics, core microbiome, differential abundance, and co-occurrence network analyses. Harvest date, finishing weight, and GIT location had a significant impact on microbial diversity and community composition (*P* < 0.05), and there was an interaction between GIT location and harvest date (*P* < 0.05). Taxonomic composition shifted throughout the GIT, though *Prevotella* and *Treponema* were core members in several different GIT locations. The co-occurrence analysis revealed microorganisms potentially associated with clinical infections, with *Moryella* in the rumen and *Acinetobacter* in the hindgut emerging as the highest scoring hubs. These results suggest that the pre-harvest period may negatively impact the beef cattle GIT microbiome. Modulating the GIT microbiome during the pre-harvest period may offer an opportunity to improve food safety.

**IMPORTANCE:**

With the global rise in antimicrobial resistance and the threat of foodborne illness, determining intervention strategies prior to harvest is a promising solution. The period between transportation from the feedlot to harvest may increase the risk of foodborne illness. During this period, cattle are withheld feed to reduce gastrointestinal tract (GIT) contents during carcass dressing. Feed withdrawal has many unintended consequences, such as acidosis and an increase in GIT pathogenic bacteria, that may result in foodborne pathogens on the final product. These consequences have yet to be thoroughly investigated in dairy-beef cross cattle, which have been rising in prominence in the United States. The GIT microbiome of dairy-beef cross cattle has been scarcely characterized despite its influence on preventing the proliferation of common pathogens in the GIT. Therefore, it is necessary to determine the impacts of feed withdrawal on the GIT microbiome and its relation to foodborne illness.

## INTRODUCTION

The pre-harvest process is a period in which cattle are transported from feedlots to processing facilities and held prior to slaughter. This is a stressful period for cattle, with several factors, such as transportation, temperature, stocking density, handling, and feed withdrawal, contributing to the stress response (1–5). The compounding stress of these factors increases circulating hormones such as cortisol and glycoproteins and haptoglobin and chromogranin A that deplete muscle glycogen stores, negatively affecting meat tenderness and color (2). In addition to the effects on meat quality, the gastrointestinal tract (GIT) microbiome can be affected by several of these factors, including feed withdrawal (6, 7). Cattle typically undergo a varying feed withdrawal period during the pre-harvest process to reduce GIT contents during carcass dressing (8, 9). Despite the advantages during harvest, feed withdrawal has been associated with acidosis, increased fecal pathogen shedding, and inflammation (9). Starvation during feed withdrawal diminishes populations of beneficial bacteria while encouraging the growth of acid-producing bacteria, such as *Streptococcus*, and pathogens, such as non-typhoidal *Salmonella enterica* (6, 7, 10, 11). This poses major challenges for the beef industry as acidosis has been linked to the shedding of fecal pathogens that can spread between animals (12, 13). Additionally, these conditions disrupt gut barrier function, which may contribute to the stress and inflammatory response (14, 15). As of late, there have been few studies measuring the impact of feed withdrawal on cattle during the pre-harvest process.

Dairy-beef crossbred cattle are rising in prevalence in the United States and present a major economic benefit to dairy farmers (16). Crossbred surplus calves have higher economic value than purebred Holstein calves, providing an additional source of income to dairy farmers while improving pregnancy rates and reducing the number of replacement heifers (17). In addition to the economic and practical benefits to dairy farmers, dairy-beef crossbred cattle, particularly Holstein × Angus crossbred cattle, have higher marbling and consumer satisfaction than purebred Holstein cattle (16, 18). Despite the benefits, dairy-beef crossbred cattle are more prone to acidosis and subsequent liver abscesses than purebred beef cattle (16, 19). Foraker et al. (16) reported that dairy-beef crossbred cattle have a 40–60% intermediate abscess rate compared to 15–30% in purebred beef cattle. This suggests that the rumen and lower GIT structure and microbial communities are more sensitive to high grain diets and potentially the stress of feed withdrawal and other pre-harvest factors (16, 20).

Of the studies examining the impact of the pre-harvest process and feed withdrawal on bovine health and food safety, few have focused on the entire GIT microbiome or Holstein × Angus crossbred cattle (Young et al., 2024). As the whole GIT is exposed to the carcass during harvest, and each GIT compartment has a unique impact on host function, understanding the effects of these microbial communities offers an opportunity to partially mitigate food safety and meat quality concerns (9, 21). Despite considerable research into these effects, few studies have ventured beyond the rumen. Given the known relationship between the small intestine's microbial communities and the stress and inflammatory responses, the small intestines are a prime target for modulating the stress from external factors (22). In addition, the hindgut microbial communities influence food safety as fecal pathogen shedding is a major source of pathogen spreading and carcass contamination (23, 24). To begin parsing the impacts of each GIT microbial community during the pre-harvest process, this study aims to characterize the GIT microbiome of Holstein × Angus crossbred cattle at harvest and begin identifying potential opportunities to improve carcass quality and food safety.

## MATERIALS AND METHODS

### Sample collection

**Fig 1:**
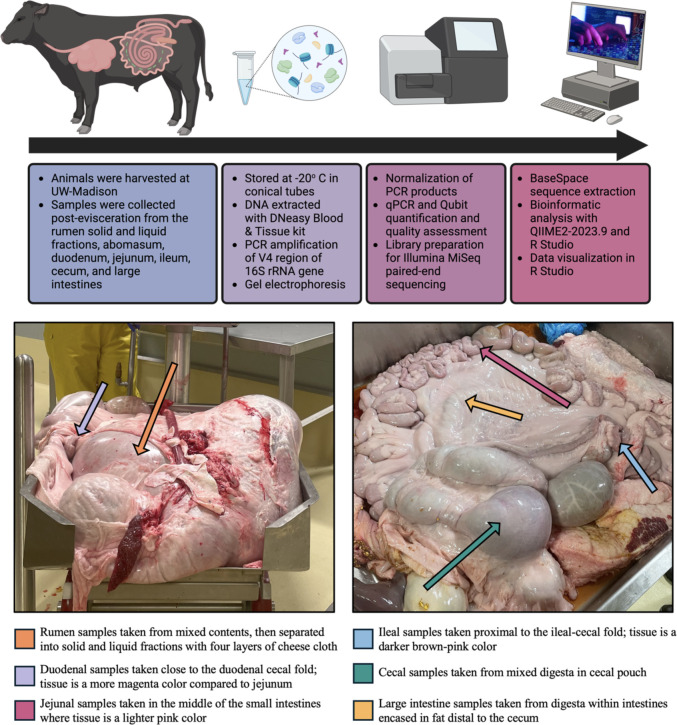
Experimental overview and sampling techniques. Figure made with Biorender (agreement number ML27HFY3SN).

Nine Holstein × Angus crossbred cattle were shipped from a single producer based in Columbia County, WI, around 20 months of age, to the University of Wisconsin-Madison (UW-Madison) USDA processing facility in the Meat Science & Animal Biologics Discovery building. From six months of age to the night before harvest, animals were housed in concrete feedlots with shelter. Animals were fed a grassy hay and corn diet mixed with the Gain Master 55:35 RT #1707 pellet (Big Gain Inc., Mankato, MN) at a 5% inclusion rate. Cattle were withheld feed and loaded onto the trailer overnight, for approximately 10 hours, and were transported to UW-Madison the morning of harvest. The transportation time from the producer to UW-Madison was one hour, and animals were held for between one and three hours at the processing facility. Nine animals were harvested on 4/6/23 (*n* = 2), 4/25/23 (*n* = 2), 6/8/23 (*n* = 3), and 8/10/23 (*n* = 2) according to USDA specifications. The average finishing weight for each harvest date is as follows: 802.7 kg on 4/6/23, 624.5 kg on 4/25/23, 659.1 kg on 6/8/23, and 615.7 kg on 8/10/23. Harvest began at approximately 7:30 AM each morning, and the average temperatures the week before harvest were as follows: 8.89°C on 4/6/23, 11.67°C on 4/25/23, 22.22°C on 6/10/23, and 22.78°C on 8/10/23. Digesta content samples were taken post-evisceration from seven locations throughout the GIT: rumen, abomasum, duodenum, jejunum, ileum, cecum, and large intestines. Due to the low volume of digesta content throughout the GIT, samples were collected based on visual identification of the compartments (as shown in Fig. 1). Immediately following collection, rumen samples were separated into solid and liquid fractions using four layers of bleached cheesecloth (Grainger Industrial Supply Lake Forest, IL, USA). Samples were stored in 50 mL conical tubes (Eppendorf, Hamburg, Germany) at −20°C before DNA extractions.

### 16S rRNA gene sequencing

Before DNA extractions, frozen samples were thawed, and 325 mg was aliquoted into 2 mL microcentrifuge tubes (Eppendorf, Hamburg, Germany). A Qiagen DNeasy Blood & Tissue Kit (Qiagen, Hilden, Germany) was used to extract DNA from the samples with a 10 minute bead beating step. DNA concentrations were quantified with a Nanodrop 1000 spectrophotometer (Thermo Fisher Scientific, Waltham, MA, USA), and samples with DNA concentrations above 15 ng/µL were diluted to 10 ng/µL in AE buffer, while samples with concentrations below 15 ng/µL were not. Following DNA extraction and dilution, the V4 region of the 16S rRNA gene region was amplified with a high-fidelity polymerase (Accuprime Pfx DNA polymerase; Thermo Fisher Scientific, Waltham, MA, USA) and dual-indexed primers, with eight nucleotide barcode sequences as developed by Kozich et al. (25). The PCR products were confirmed using gel electrophoresis, and successfully amplified products were normalized to 20 nM with a SequalPrep Normalization kit (Life Technologies, Carlsbad, CA, USA). Final libraries were created with 5 µL of each of the normalized samples. The final library DNA concentrations were determined with an Illumina platform-specific KAPA library quantification kit (Kapa Biosystems, Inc., Wilmington, MA, USA) and 1× High Specificity Assay kit on a Qubit 4 fluorometer (Invitrogen, Carlsbad, CA, USA). The pooled library was then diluted to 20 nM and combined with HT1 buffer, 20 nM of PhiX v3 control, and 0.2 N NaOH to provide a final concentration of 6 pM. The sample solution was mixed with PhiX control v3 (20%, vol/vol) before 600 µL was loaded into a MiSeq v2 (500 cycles) reagent cartridge (Illumina, San Diego, CA, USA).

The sequences were downloaded from Illumina BaseSpace. The sequences were subsequently downloaded locally and input into QIIME2-amplicon-2023.9 via the Casava1.8 paired-end pipeline (26). Amplicon sequencing variant (ASV) taxonomic assignment was completed with classify-sklearn and the SILVA 2023.9 database with a confidence limit of 95%. After visualization, the sequences were trimmed with DADA2 in the chimera consensus pipeline (27). The taxonomic output file, sample metadata, rooted phylogeny tree, and feature table were uploaded into R Studio for further statistical analyses.

### Statistical analyses

Several packages were used for statistical analysis and visualization, including Phyloseq (McMurdie and Holmes, 2013), qiime2R (Biasanz, 2018),(28) DEseq2 (29), vegan (30), microbiomeutilities (31), and SpiecEasi (32). Using linear regression models, we analyzed and visualized alpha diversity for diversity and richness with Pielou's evenness, observed features, Simpson's index, Chao index, and Shannon's diversity index. These models were assessed for normality with the Shapiro test, the Lilliefors test, the Cramér–von Mises test, and the Anderson-Darling test. The Akaike's information criterion (AICcmodavg) was implemented on each potential model, and the model with the lowest AIC was selected (alpha diversity = harvest date + GIT location + Finishing wt + harvest date*GIT location). An analysis of variance (ANOVA) test was completed to determine group significance and interactions. Alpha diversity pairwise comparisons were analyzed using Tukey's honest significant differences test. Beta diversity was assessed with two quantitative indicators, the Bray-Curtis dissimilarity index and the weighted Unifrac distance matrix, considering both variation and population dispersion with the analysis of similarity (ANOSIM) function. Core microbial members were identified with a core microbiome analysis (microbiomeutilities) with a detection setting, the minimum relative abundance of a taxa, of 0.01 and a prevalence of 20% due to high sample variability (31). Differential abundance analyses were completed with the DESeq2 package, which uses an analysis of the compositional profiles of microorganisms (ANCOM) and a Wald test at 0.0001 (29). Co-occurrence networks were determined and visualized with methods described by Amorín de Hegedüs et al. and the mdmnets package (33). Due to the low sample size, GIT locations within a compartment were analyzed together for the differential abundance analysis and the co-occurrence network analysis. The rumen (rumen solids and rumen liquids), the small intestines (duodenum, jejunum, and ileum), and the hindgut (cecum and large intestines) were pooled to create three locations for these analyses. Data were visualized with ggplot2 and RColorConesa.

## RESULTS

### Microbial diversity varied across the GIT and between harvest dates

**Fig 2:**
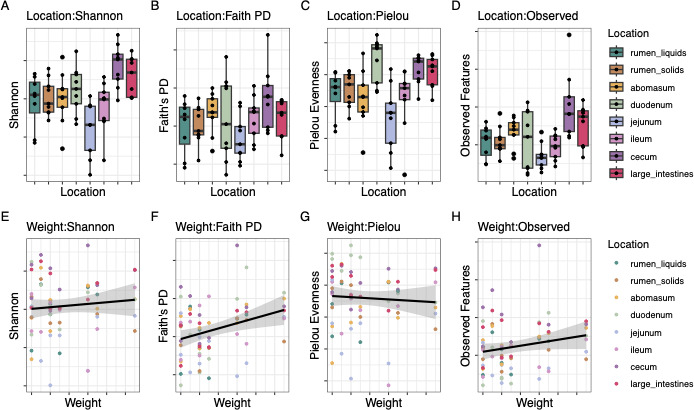
Microbial diversity (A, E: Shannon's diversity; B, F: Faith's phylogenetic diversity; C, G: Pielou's evenness; D, H: observed features) between location (**A, B, C, D**) and finishing weight (**E, F, G, H**) with a 95% confidence interval and standard error.

**Fig 3:**
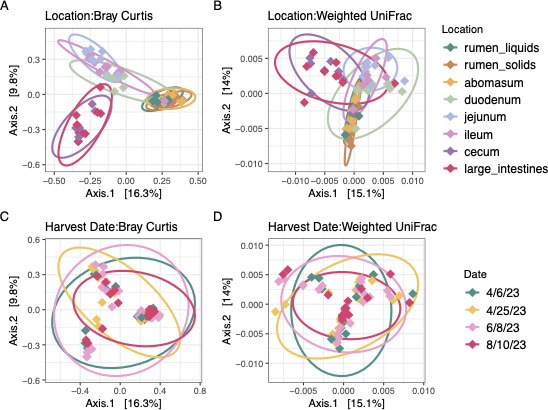
Community dissimilarity (A, C: Bray-Curtis; B, D: weighted Unifrac) between GIT location (**A, B**) and harvest date (**C, D**).

In this study, we aimed to characterize the microbiome throughout the GIT at harvest. The effects of GIT location, harvest date, and the interaction between both variables on sample evenness and richness were assessed with ANOVA (Table 1), and the phylogenetic diversity and abundances of each sample were determined with ANOSIM considering these factors and their combinatory effect (Tables 1 and 2). Gastrointestinal tract location was the strongest indicator of microbial diversity (*P* < 0.05; Tables 1 and 2) and community dissimilarity (*P* < 0.05; Table 1; Fig. 3A and B). The cecum had the highest richness and evenness compared to the other GIT locations, while the jejunum had the lowest richness (*P* < 0.05). Finishing weight had a significant impact on microbial diversity and community structure (*P* < 0.05; Tables 1 and 2; Fig. 2E through H). Harvest date also had a significant impact on sample richness and evenness (*P* < 0.05; Tables 1 and 2; Fig. 2A through D) and community composition (*P* < 0.05; Tables 1 and 2; Fig. 3C and D). Cattle harvested on 4/25/23 (*n* = 2) had the highest microbial diversity of any harvest date and significantly higher richness and evenness than cattle harvested on 8/10/23 (*n* = 2), the harvest date with the lowest diversity (*P* < 0.05; Fig. 2A through D). Community composition, sample richness, and evenness were also impacted by an interaction between GIT location and harvest date (Tables 1 and 2). Each GIT compartment exhibited a distinct community structure (*P* < 0.05), though the GIT locations within each compartment did not (*P* < 0.05). Therefore, the sampling locations within each GIT compartment were pooled for the differential abundance and community network analyses.

**TABLE 1:** ANOVA results for each alpha diversity metric

Factor	Shannon	Simpson	Chao1	Observed	Pielou	Faith
Date	0.00021*^a^	0.10	0.000000239*	0.0000022*	0.474	0.0000214*
Location	0.00000086*	0.0023*	0.00000032*	0.00000031*	0.00000076*	0.0069*
Weight	0.21	0.154	0.46	0.0034*	0.036*	0.00021*
Date*Location	0.04*	0.31	0.051	0.04^*^	0.0013*	0.068

^*a*^ *P < 0.05.

**TABLE 2:** ADONIS results for each beta diversity metric

Factor	Bray	Jaccard	Unweighted Unifrac	Weighted Unifrac
Date	0.01*^a^	0.01*	0.001*	0.001*
Location	0.01*	0.01*	0.001*	0.001*
Weight	0.01*	0.01*	0.007*	0.001*
Date × location	0.03*	0.02*	0.09	0.049*

^*a*^ *, P < 0.05.

### Community composition differed across the GIT

**Fig 4:**
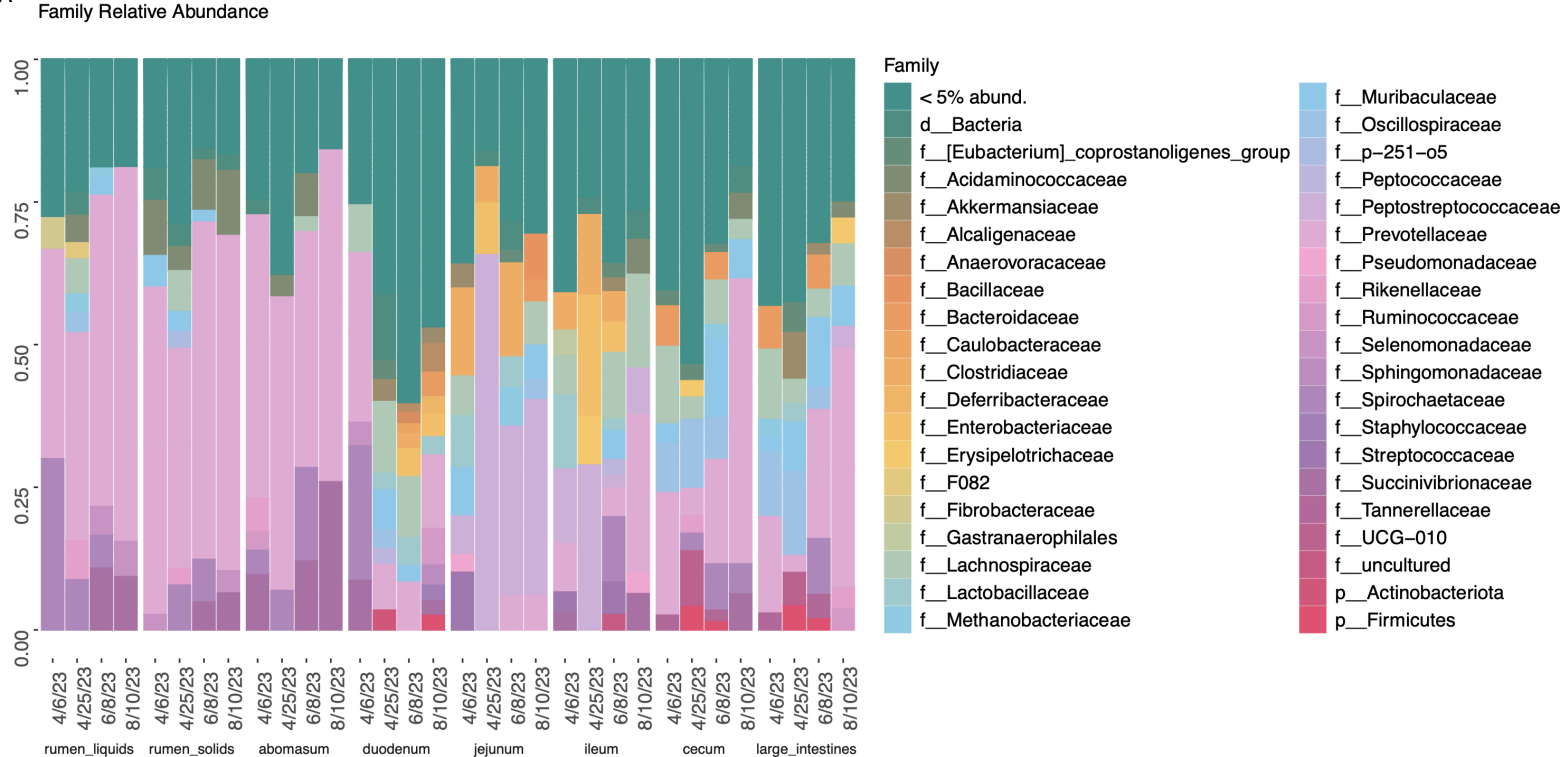
Taxonomic bar plots of family level relative abundances.

**Fig 5:**
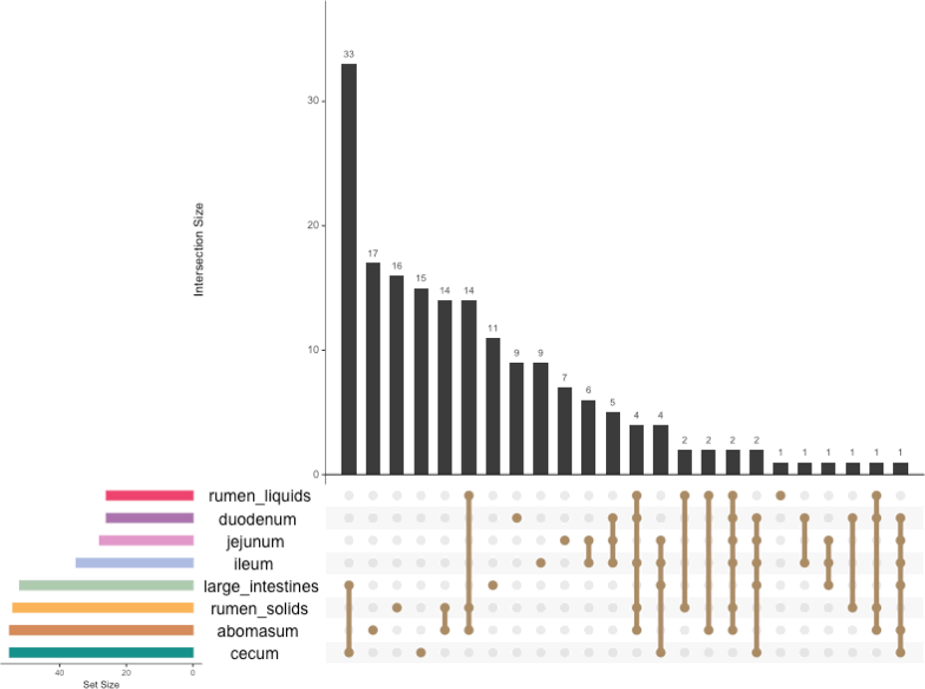
Shared ASVs between GIT compartments. Amplicon sequencing variants were considered core members if they were higher than 0.01% abundance and detected in at least 50% of the samples. The total number of core members in each location is shown on the left, and the number of shared ASVs is visualized as intersection size.

Community structure shifted across the GIT, with numerous shared taxa between neighboring GIT locations (Fig. 4 and 5). The top 40 most abundant taxa analysis was completed alongside a core microbiome analysis, with a prevalence of 50% and 0.1% detection, to determine microbial community structure (Tables S1 and S2). The rumen liquid and solid fractions had similar top taxa and core members. *Prevotellaceae* YAB2003*, Prevotella, Succinivibrionaceae* UCG-001*, Treponema, Muribaculaceae,* and *Rikenellaceae* RC9 gut group were among the most abundant in the rumen liquids and solids. These listed taxa were also shared core members between the rumen liquids and solids (Fig. 5). There was minimal methanogen representation in the rumen, with only *Methanobrevibacter* as the 23rd most abundant taxa in the rumen solids fraction. The top taxa and core members in the rumen were nearly identical in the abomasum and included *Prevotella, Succinivibrionaceae* UCG-001*,* and *Treponema*.

The lower GIT shared numerous highly abundant and core taxa. In the duodenum, *Prevotella, Lachnospiraceae, Muribaculaceae, Pseudomonas,* and *Lactobacillus* were considered core members (Table S2; Fig. 5). Several bacteria, *Akkermansia. Peptostreptococcaceae*, *Clostridium senso stricto 1, Lactobacillus,* and Enterobacteriaceae, were identified as the most prevalent taxa in the jejunum and ileum. While not a core member, *Staphylococcus* was represented in the top 40 most abundant taxa in the jejunum and ileum (Table S1). The duodenum, jejunum, and ileum shared many core taxa, including *Turicibacter*, *Methanobrevibacter, Akkermansia,* and *Clostridium senso stricto* 1. As observed in the small intestines, the cecum and large intestines shared many top taxa, including *Alloprevotella, Prevotella,* and *Clostridia*. There were 55 core members in the cecum, including *Methanobrevibacter, Clostridium senso stricto* 1, *Muribaculaceae*, and *Oscillospiraceae* UCG-005 (Table S2; Fig. 5). The large intestines shared many of their 52 core taxa with the cecum, except for *Treponema, Succinivibrio,* and several *Alloprevotella* taxa. In contrast to the rumen fractions, two methanogens were among the top 40 most abundant taxa in the hindgut, including *Methanobrevibacter* and *Methanocorpusculum*.

### Low-abundant taxa were critical to community structure in each GIT compartment

**Fig 6:**
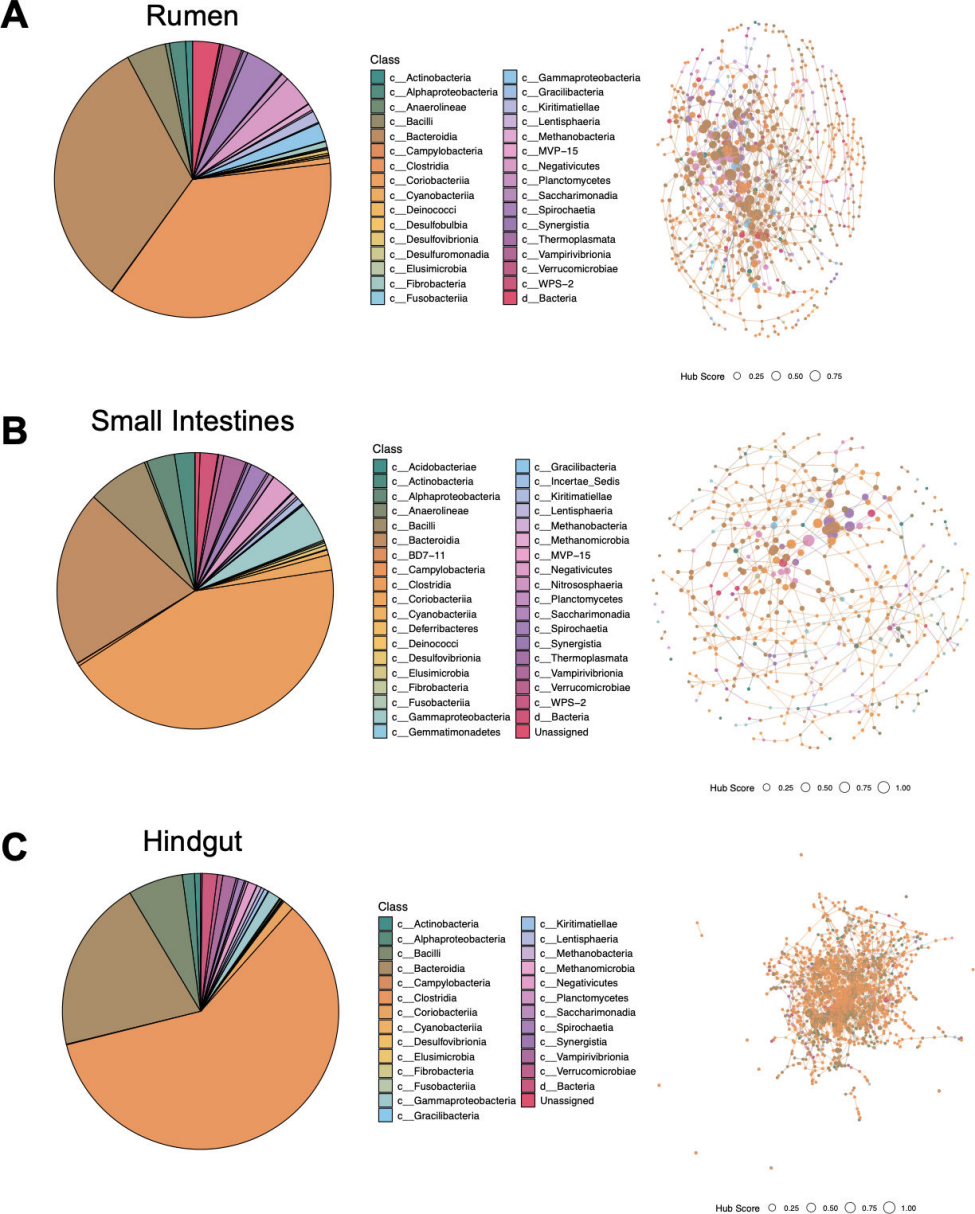
Co-occurrence networks for the rumen (rumen solids and liquids; *n* = 18) (**A**), small intestines (duodenum, jejunum, and ileum; *n* = 27) (**B**), and hindgut (cecum and large intestines; *n* = 18) (**C**). The distribution of class in the co-occurrence networks is visualized in the pie charts. Hub networks at the class level are shown for each compartment. High-scoring hubs are represented by a larger dot while low-scoring hubs are represented by a smaller dot, as shown in the legend.

Co-occurrence networks were built for each GIT compartment: the rumen (rumen solids and rumen liquids; *n* = 18; Fig. 6A), small intestines (duodenum, jejunum, and ileum; *n* = 27; Fig. 6B), and hindgut (cecum and large intestines; *n* = 18; Fig. 6C). Compartments were pooled due to insignificant differences between community composition within compartment (*P* < 0.05) and to increase sample size for the co-occurrence network analysis. A co-occurrence network is analyzed and assessed with the determination of three factors: degree of centrality, closeness centrality, and betweenness centrality (34). Hub scores are assigned, taking into consideration all three of these metrics, and a hub score of 1 indicates a keystone member in an ecosystem. All 25 of the highest hub scores in the rumen and small intestines had hub scores above 0.7 (Table 3). However, 19 out of the 25 highest scoring hubs were below 0.7 in the hindgut. *Moryella* was the highest scoring hub (1.00) in the rumen despite having relatively low abundance in both fractions, and *Megasphaera* was the second highest scoring hub (0.963). In the small intestines, *Muribaculaceae* had the highest hub score (1.00). While several *Muribaculaceae* taxa were considered core members across the locations within the small intestines, the taxa identified as keystone members were not. Three core members in the small intestines, *Methanobrevibacter* (0.778), *Micrococcaceae* (0.928), and *Prevotella* (0.962), were represented in the top 25 highest scoring hubs in the small intestines, alongside several less predominant taxa, including *Eubacterium coprostanoligenes* group (0.831) and *Streptococcus* (0.829). Interestingly, *Acinetobacter* (1.00) was the highest scoring hub in the hindgut despite not being in the top 40 most abundant taxa of the cecum or large intestines, with no representation in the core microbiome analysis. *Christensenellaceae* R7 group (0.782)*, Pyramidobacter* (0.729), *Roseburia* (0.649), and *Defluvitaleaceae* UCG-001 (0.645) were among the least abundant genera in the top 25 highest hub score groups in the hindgut.

**TABLE 3:** Top 25 highest scoring hubs in each GIT compartment: rumen (rumen solids and liquids), small intestines (duodenum, jejunum, and ileum), and hindgut (cecum and large intestines)

Compartment	Hub score	Genus
Rumen	1.000	*Moryella*
Rumen	0.963	*Megasphaera*
Rumen	0.919	*Clostridia*
Rumen	0.890	*Lachnospiraceae*
Rumen	0.878	*Clostridia*
Rumen	0.851	*Lachnospiraceae_NK3A20_group*
Rumen	0.846	*Prevotella*
Rumen	0.831	*Prevotellaceae_YAB2003_group*
Rumen	0.829	*Prevotella*
Rumen	0.815	*Lachnospiraceae*
Rumen	0.813	*Lachnospiraceae_NK3A20_group*
Rumen	0.793	*Lachnospiraceae*
Rumen	0.790	*Prevotella*
Rumen	0.781	*Bacteroidales*
Rumen	0.779	*Proteobacteria*
Rumen	0.778	*Rikenellaceae_RC9_gut_group*
Rumen	0.771	*Prevotellaceae*
Rumen	0.771	*Bacteroidales*
Rumen	0.760	*Spirochaetaceae*
Rumen	0.758	*Bacteroidales*
Rumen	0.754	*Elusimicrobium*
Rumen	0.750	*Oribacterium*
Rumen	0.729	*Muribaculaceae*
Rumen	0.726	*Prevotellaceae_UCG-004*
Rumen	0.726	*Lachnospiraceae*
Small intestines	1.000	*Muribaculaceae*
Small intestines	0.962	*Prevotella*
Small intestines	0.933	*Lachnospiraceae*
Small intestines	0.928	*Micrococcaceae*
Small intestines	0.879	*NK4A214_group*
Small intestines	0.864	*Bacteroidales*
Small intestines	0.831	*[Eubacterium]_coprostanoligenes_group*
Small intestines	0.829	*Streptococcus*
Small intestines	0.823	*Prevotella*
Small intestines	0.821	*Lachnospiraceae*
Small intestines	0.814	*Akkermansia*
Small intestines	0.805	*Bacteroides*
Small intestines	0.798	*Prevotella*
Small intestines	0.796	*F082*
Small intestines	0.787	*Prevotella*
Small intestines	0.784	*Enterobacterales*
Small intestines	0.784	*Chloroplast*
Small intestines	0.781	*UCG-005*
Small intestines	0.778	*vadinBE97*
Small intestines	0.778	*Methanobrevibacter*
Small intestines	0.777	*Lachnospiraceae*
Small intestines	0.776	*F082*
Small intestines	0.768	*Lachnospiraceae*
Small intestines	0.767	*Mitochondria*
Small intestines	0.760	*Bacteroides*
Hindgut	1.000	*Acinetobacter*
Hindgut	0.864	*Lachnospiraceae*
Hindgut	0.855	*UCG-005*
Hindgut	0.782	*Christensenellaceae_R-7_group*
Hindgut	0.729	*Pyramidobacter*
Hindgut	0.724	*Lachnospiraceae*
Hindgut	0.692	*Clostridia_UCG-014*
Hindgut	0.678	*Muribaculaceae*
Hindgut	0.674	*Clostridia*
Hindgut	0.664	*Enterobacterales*
Hindgut	0.656	*Peptostreptococcaceae*
Hindgut	0.654	*UCG-010*
Hindgut	0.648	*Gastranaerophilales*
Hindgut	0.646	*Roseburia*
Hindgut	0.645	*Defluviitaleaceae_UCG-011*
Hindgut	0.640	*Lachnospiraceae*
Hindgut	0.640	*Lactobacillus*
Hindgut	0.637	*Clostridium_sensu_stricto_6*
Hindgut	0.637	*UCG-010*
Hindgut	0.632	*Lactobacillus*
Hindgut	0.629	*[Eubacterium]_coprostanoligenes_group*
Hindgut	0.623	*UCG-009*
Hindgut	0.621	*Lachnospiraceae*
Hindgut	0.592	*Peptostreptococcaceae*
Hindgut	0.589	*Prevotella*

### The harvest dates with the most and least diverse GIT microbiomes had many differentially abundant taxa

**Fig 7:**
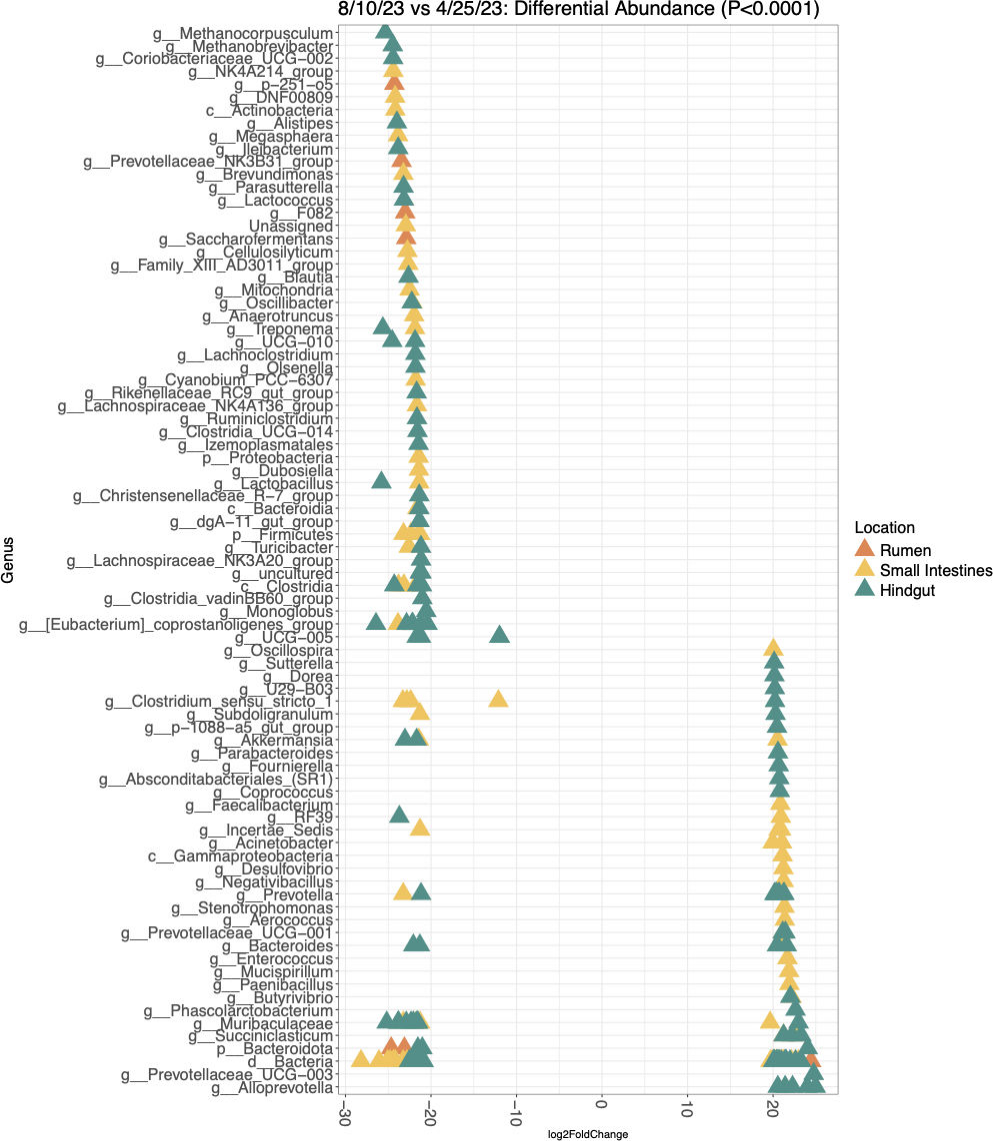
Differential abundance analysis of 8/10/23, the least diverse sampling date, vs. 4/25/23, the most diverse sampling date, on a log-fold change scale for each GIT location. A negative log-fold change indicates taxa enriched on the 4/25/23 timepoint, while a positive log-fold change is enriched on 8/10/23. These two harvest dates had the most significant differences in microbial diversity (*P* = 0.0058). Taxa were considered differentially abundant at *P* < 0.0001 due to low sampling size. An analysis was performed on all GIT compartments: foregut (rumen solids and liquids) (*n* = 4 per harvest date), small intestines (duodenum, jejunum, and ileum) (*n* = 6 per harvest date), and hindgut (cecum and large intestines) (*n* = 4 per harvest date).

Harvest date affected community structure within the foregut (rumen solids and liquids, *n* = 4), small intestines (duodenum, jejunum, and ileum; *n* = 6), and hindgut (cecum and large intestines, *n* = 4). Only the least and most diverse harvest dates were compared to demonstrate potential differences between animals with more and less diverse GIT microbiomes. The differences between the most diverse harvest date, 4/25/23, and the least diverse harvest date, 8/10/23, were analyzed by GIT compartment (Fig. 7). These results are shown in Fig. 7. In the rumen, several *Prevotella* ASVs, *Methanobrevibacter, Fibrobacter*, and *Bacteroidota* were enriched on 8/10/23. There were 93 differentially abundant taxa in the small intestines, including many classified as family *Prevotellaceae, Lachnospiraceae,* and *Rikenellaceae*. There were three methanogenic ASVs enriched in the small intestines on 4/25/23, two classified as *Methanobrevibacter* and *Methanocorpusculum*. These taxa were also enriched in the hindgut on 4/25/23, along with *Eubacterium coprostanoligenes* group, *Clostridia* UCG-014, and *Desulfovibrio*.

## DISCUSSION

The GIT microbiome is central to beef cattle production and food safety (8, 35). The rumen and fecal microbiome have been tied to the prevention of pathogen colonization and host immune response (35). Under normal conditions, the collective rumen microbiome prevents the rapid growth and domination of harmful bacteria, such as *Salmonella* or *F. necrophorum* (6, 7). However, during starvation conditions, beneficial bacteria die off and opportunistic pathogens rapidly proliferate (12). The small intestines and hindgut microbial communities function similarly without stressors, harboring populations of bacteria, such as *Succinivibrio, Prevotella,* and *Butyrivibrio,* that promote mucus secretion, blocking pathogenic colonization (35, 36). The absence of these beneficial bacteria can shift dominance to mucus-degrading communities that stimulate inflammatory gene expression in the gut epithelium, contributing to systemic inflammation (22, 37). In the hindgut, acidosis can damage the epithelium and promote pathogen proliferation (13). Furthermore, bacteria that are normally beneficial may become functionally harmful during dysbiosis. For instance, certain *Clostridium* and *Escherichia* species may occupy a small niche during high substrate availability, becoming opportunistic when competing bacteria are eliminated (38, 39). A healthy and responsive GIT microbiome is important during pre-harvest transportation and lairage period to reduce adverse effects from stress and feed withdrawal (35, 40).

The current study surveyed the GIT microbiome of nine beef cattle entering a small USDA processing facility from a single producer on four different dates. The GIT microbiome varied between harvest dates, as shown by the significant differences in microbial diversity and community composition. For instance, cattle harvested during the first three harvest dates had significantly higher microbial diversity than cattle harvested on 8/10/23, and there were upwards of 100 differentially abundant taxa between the most diverse harvest date, 4/25/23, and the least diverse harvest date in the hindgut. The low number of animals harvested on each date prevents broad conclusions; however, there are numerous factors that may have contributed to the observed differences between harvest dates. An overnight feed withdrawal period, which was estimated to be approximately 10 hours, was reported by the producer. Given the USDA processing facility's overnight feed withdrawal guidelines and the lack of strict requirements, it is possible that some of the feed withdrawal estimations varied by several hours. Higher finishing weight was also associated with higher phylogenetic diversity and lower community evenness. In this study, animals were reportedly slaughtered around 20 months of age, and therefore, heavier animals were likely to be more feed efficient. Several studies have linked feed efficiency with resilience, which may have a positive impact on microbial diversity (41–43). In addition to potentially inconsistent feed withdrawal periods, there were major differences in temperature between harvest dates. The first two harvest dates, 4/6/23 and 4/25/23, had lower average weekly temperatures than the latter two harvest dates, 6/8/23 and 8/10/23. It has been previously reported that both feed withdrawal and high temperatures negatively affect the GIT microbiome (6, 7, 38). Both conditions increase the abundance of lactic acid-producing bacteria in the rumen, which lowers ruminal pH and disrupts regular gut barrier function (6, 7, 38). Thus, more studies are necessary to explore the effects of different pre-harvest factors on the GIT microbiome and compare the microbial effects to food safety and animal welfare concerns.

The pre-harvest GIT microbiome networks and composition harbored taxa related to inflammation. *Megasphaera*, a lactic acid-utilizing bacteria, and *Streptococcus*, a lactic-acid-producing bacteria, were high-scoring hubs in the rumen, which may contribute to acidosis (6, 44). Ruminal acidosis can directly affect the inflammatory response as the osmotic pressure from acidic contents can damage the rumen epithelium, promoting inflammatory gene expression (Zhao et al., 2019; Baaske et al., 2019). Additionally, the antagonistic association between acidic conditions and bacterial diversity and richness can promote pathogen proliferation (45, 46). The enriched abundance of certain pathogens can lead to an accumulation of bioamines and lipopolysaccharides that can further aggravate the epithelium (45, 46). In the small intestines, *Streptococcus* and *Muribaculaceae* were high-scoring hubs as well as core members. *Muribaculaceae* is a known mucus degrader and increases in abundance during fasting periods (47, 48). This trend continued through the large intestines, where *Muribaculaceae* and *Parabacteroides* were core members. While typically reported as a commensal taxon, *Parabacteroides* has been associated with disease and chronic inflammation in humans under stress conditions (36, 49). Meat quality is negatively affected by increased inflammation, as inflammation can elevate cortisol levels (2, 50). As animals are simultaneously experiencing increased stress from external pre-harvest factors, the compounded inflammation from the GIT only exacerbates meat quality concerns. Therefore, pro-inflammatory taxa that affect gut barrier function may increase pathogen migration from the GIT (51).

Several potentially pathogenic and spoilage bacteria were identified throughout the GIT. *Moryella,* the highest hub score in the rumen and a keystone genus in the community network, has previously been associated with mastitis and *E. coli* O157:H7 fecal shedding in dairy cattle (3, 52). The association between *Moryella* and pathogenesis is likely due to its involvement in indole production, a signaling molecule that may affect bacterial virulence and LPS production, which contributes to host inflammation (52, 53). *Moryella* can also migrate from the GIT into pus, which may exacerbate the stress response by penetrating the gut barrier in compromised pre-harvest animals (52). The second most abundant taxa in the small intestines, *Clostridium senso stricto 1* (a taxonomic group including *Clostridium perfringens*)*,* has been associated with diarrhea in calves (4). Additionally, *Pseudomonas*, a potentially opportunistic pathogen and spoilage organism, was a core member of the microbial community in the ileum (54, 55). The highest scoring hub in the hindgut, *Acinetobacter,* is commonly present on beef, and there have been several reports of antimicrobial resistance genes in *Acinetobacter* isolates from beef samples (56–58). When considering the intersection of pro-inflammatory, mucus-degrading bacteria and pathogenic proliferation, the host may be more prone to stress and systemic infection (51). For instance, a higher prevalence of acid-producing bacteria can induce an epithelial inflammatory response and increase gut permeability (51). Increased gut permeability is advantageous for certain pathogens that can migrate from the GIT into the bloodstream (59). For instance, *F. necrophorum* is highly associated with ruminal acidosis due to the increased opportunity to migrate from the rumen to the liver and form abscesses (59). Therefore, understanding the impact of the microbial communities throughout the GIT is critical to modulate the negative effects of pre-harvest factors on food safety and meat quality.

The prevalence of dairy-beef cross cattle has been rapidly increasing, as reported by a 200% increase in the five-year average of beef semen sales in 2020 and presents significant economic benefits to dairy producers (16). Despite this, dairy-beef crosses have a high prevalence of liver abscesses (19). Liver abscesses have been associated with a decrease in average daily gain and hot carcass weight (60). Therefore, while dairy-beef crosses offer significant economic benefits, they simultaneously present food safety concerns. In the present study, *Bacteriodes* was a core member in the duodenum, cecum, and large intestines, as well as the fifth most abundant genus in the cecum. *Bacteriodes* has previously been identified in the liver abscess microbiome with *F. necrophorum*, supporting the hypothesized link between the GIT microbiome and liver abscess prevalence (60, 61). *Acinetobacter* has also been identified in the liver abscess microbiome, specifically in cattle fed tylosin phosphate (62, 63). Currently, tylosin phosphate, Tylan, is a popular feed additive in finishing diets to reduce liver abscesses. The cattle in this study were fed the Gain Master 55:35 RT #1707 pellet at a 5% inclusion rate, which contains Tylan (160 g/ton) to prevent liver abscesses. Pinnell et al. observed an increased abundance of *Succinivibrionacae* UCG-001 in the rumen and *Turicibacter* in the colon epithelium when feeding tylosin (61), similar to digesta microbiome findings of this study. Despite reducing liver abscesses, tylosin has been observed to select for macrolide-resistant bacteria, posing a major food safety risk as common foodborne pathogens, such as *Enterococcus* species, *E. coli, Campylobacter*, and *Salmonella*, develop genetic resistance (64, 65). With growing concerns relating to liver abscess prevalence and antimicrobial resistance, modulating the microbiome has become a more desirable approach to controlling microbial pathogens in the GI tract of ruminants (61, 64, 65). Therefore, future studies will be necessary to link the GIT microbiome at harvest with liver abscesses and antibiotic usage to determine future mitigation methods that reduce economic losses and improve food safety, especially in dairy-beef crossbred cattle.

The results of this study demonstrate future research strategies in harnessing the GIT microbiome to limit the welfare, meat quality, and food safety consequences of the pre-harvest process, especially in Holstein × Angus crossbred cattle. For instance, protecting or modifying the rumen microbiome prior to harvest to improve resiliency may reduce the risk of liver abscesses or inflammation during feed withdrawal (59, 66). The small intestinal epithelium has a high concentration of immune-related cells, and by reducing the abundance of pro-inflammatory taxa under starvation conditions, such as *Streptococcus* or *Pseudomonas,* animals may experience less systemic inflammation (67). Several studies have previously identified certain taxa in the feces, such as *Moryella* and *Clostridium,* to be associated with *E. coli* O157:H7 shedding in cattle (3, 68). Finally, while feed withdrawal has several positive benefits during the harvest process, its potential negative effects on the GIT microbiome, inflammation, and food safety may need further consideration. Additional research is necessary to better evaluate the pros and cons of feed withdrawal to balance ease of processing with food safety. The results of this study offer insight into the state of the entire cattle GIT microbiome at harvest.

## Data Availability

The raw data were deposited in the NCBI BioProject database under accession number PRJNA1204970.

## SUPPLEMENTAL MATERIAL

Tables S1 to S3 aem.02599-24-s0001.xlsx Tables illustrating the top 40 genera of each location, core microbiome, and differentially abundant taxa.

ASM does not own the copyrights to Supplemental Material that may be linked to, or accessed through, an article. The authors have granted ASM a non-exclusive, world-wide license to publish the Supplemental Material files. Please contact the corresponding author directly for reuse.
